# An 8.8 ps RMS Resolution Time-To-Digital Converter Implemented in a 60 nm FPGA with Real-Time Temperature Correction

**DOI:** 10.3390/s20082172

**Published:** 2020-04-11

**Authors:** Zhipeng Song, Zhixiang Zhao, Hongsen Yu, Jingwu Yang, Xi Zhang, Tengjie Sui, Jianfeng Xu, Siwei Xie, Qiu Huang, Qiyu Peng

**Affiliations:** 1 State Key Laboratory of Digital Manufacturing Equipment and Technology, School of Mechanical Science and Engineering, Huazhong University of Science and Technology, Wuhan 430074, China; m201970747@hust.edu.cn (Z.S.); m201970550@hust.edu.cn (H.Y.); jingwuyang@hust.edu.cn (J.Y.); xizhang@hust.edu.cn (X.Z.); stj@hust.edu.cn (T.S.); 2 School of Biomedical Engineering, Shanghai Jiao Tong University, Shanghai 200000, China; jasonorzzx@sjtu.edu.cn; 3 Pitech Company, Shenzhen 518000, China; 4 Department of Molecular Biophysics and Integrated Bioimaging, Lawrence Berkeley National Laboratory, Berkeley, CA 94720, USA

**Keywords:** time-to-digital converter (TDC), field-programmable gate arrays (FPGA), non-uniform multiphase (NUMP) method, temperature correction

## Abstract

This paper presented a non-uniform multiphase (NUMP) time-to-digital converter (TDC) implemented in a field-programmable gate array (FPGA) with real-time automatic temperature compensation. NUMP-TDC is a novel, low-cost, high-performance TDC that has achieved an excellent performance in Altera Cyclone V FPGA. The root mean square (RMS) for the intrinsic timing resolution was 2.3 ps. However, the propagation delays in the delay chain of some FPGAs (for example, the Altera Cyclone 10 LP) vary significantly as the temperature changes. Thus, the timing performances of NUMP-TDCs implemented in those FPGAs are significantly impacted by temperature fluctuations. In this study, a simple method was developed to monitor variations in propagation delays using two registers deployed at both ends of the delay chain and compensate for changes in propagation delay using a look-up table (LUT). When the variations exceeded a certain threshold, the LUT for the delay correction was updated, and a bin-by-bin correction was launched. Using this correction approach, a resolution of 8.8 ps RMS over a wide temperature range (5 °C to 80 °C) had been achieved in a NUMP-TDC implemented in a Cyclone 10 LP FPGA.

## 1. Introduction

High-resolution time-to-digital converters (TDCs) are widely used in medical time-of-flight (TOF) positron emission tomography (PET) cameras [1,2,3], light detection and ranging (LiDAR) [4,5,6], large high-altitude air shower observatory systems (LHAASO) [7,8,9], and high-energy physics (HEP) experiments [10]. Many applications have a requirement for temperature-insensitive high-performance TDCs that will provide stable and reliable operation over a wide range of temperatures.

Field programmable gate array (FPGA)-based TDCs, constructed with off-the-shelf low-cost components, offer a promising practical alternative to conventional application specific integrated circuit (ASIC)-based TDCs. Most high-resolution FPGA-based TDCs are constructed using carry chains (delay chains) in the FPGA. The performance of those FPGA-based TDCs is largely determined by the accuracy and stability of the propagation delays in the delay chain. 

The propagation delay in the delay chain is vulnerable to the influence of manufacturing processes and FPGA operating voltages and temperatures (PVT) [11,12]. In practice, variations in delay characteristics can be calibrated through code density tests [13]. Variations in propagation delay due to the operating voltage can be reduced by using high-performance power supplies [14]. However, it is not feasible or practical to control the ambient temperatures in many applications. In wave union TDC [15], a look-up table (LUT) for calculating timestamp has been built in the initial calibration mode. The LUT has been updated using the random event data in normal mode to compensate for the effects of the temperature fluctuations on propagation delays. This correction approach has achieved continuous calibration based on the technique of the ping-pang memories. However, this approach may not be suitable in applications with non-random event data inputs. Variations in propagation delays due to temperature fluctuations can be corrected using on-board LUT containing the variations of width for the time bins [16,17] or the temperature-LUT coefficient [18]. However, these methods require a temperature sensor and a regulating device and require intensive experiments to be performed to determine the relationship between the temperature and the correction parameters. The technology of dual delay lines (DDLs) is used to enable real-time calibrations in [19], which sets up an alternate result of calibrations to offset environmental effects. However, calibrating continuously results in the large power consumption, and the use of DDLs and the double backup of calibration results will consume lots of FPGA resources. A hardware unit consisting of an inverter and buffers is used to detect the ambient temperature and generate the correct values [20] but does not achieve an excellent performance.

In this paper, a novel scheme was introduced for real-time monitoring and compensation of changes in propagation delays. The event that triggered the temperature correction process was the variation of the propagation delay. The detection of the event was performed according to periodic discrete-time measurements, which could be classified as the periodic event-triggered control (PETC) [21]. When propagation delay changes exceeded a certain threshold, the bin-by-bin correction was launched, and LUT was updated on the threshold. Thus, the temperature correction scheme could be considered as an event-based control approach [22,23,24,25,26,27]. The proposed scheme was validated in a two-channel non-uniform multiphase (NUMP) TDC [28] using an Altera 60 nm Cyclone 10 LP FPGA and achieved a resolution of 8.8 ps root mean square (RMS) over a wide temperature range from 5 °C to 80 °C. 

This paper is organized as follows. In Section 2, the principle of the NUMP TDC is described briefly, and the temperature characteristics of the delay chain and the principle of the proposed temperature correction method are described. Section 3 introduces the implementation details. The experimental results are presented in Section 4, and several design key points are discussed in Section 5. Finally, Section 6 summarizes and concludes the paper.

## 2. Operating Principles

### 2.1. Top-Level Diagram of NUMP TDC

**Figure 1:**
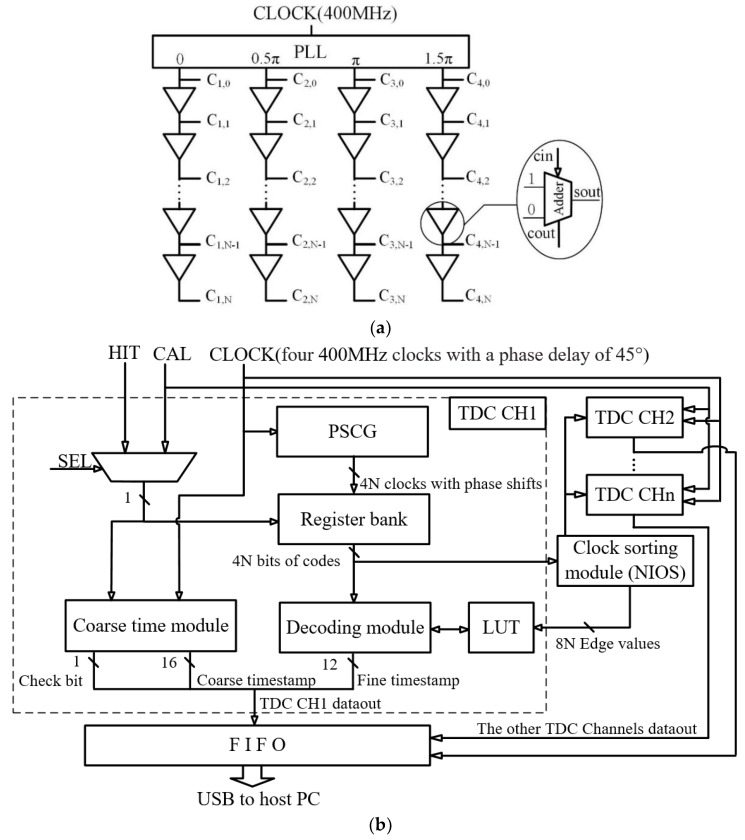
The architecture of NUMP TDC. (**a**) PSCG constructed with multiple delay chains (carry chains). (**b**) Diagram of the NUMP TDC. NUMP, non-uniform multiphase; TDC, time-to-digital converter; PSCG, phase-shifted clock generator.

The structure of the NUMP TDC is shown in Figure 1 [28]. As shown in Figure 1a, a phase-shifted clock generator (PSCG) is used to produce many copies of the system clock (400 MHz) with different phase shifts. A special structure is used to reduce the accumulation of delay chain jitter containing four equal-length sub-delay chains with a fixed phase difference (0.5π) clock feed equivalent to one delay chain. Each sub-delay chain consists of 95 delay units, and each delay unit corresponds to the carry chain of an adder. Every adder in PSCG is paired up with a dedicated register in the register bank, so there are 380 registers in the register bank. When the clock is fed into the first adder, multiple clocks with random phases are generated from the sum-out pins of the adders.

A key feature of the NUMP TDC is that it is “non-uniform”, which means that the phase relationships between the multiple clocks that are output from the PSCG are neither uniformly distributed nor sorted in time order. Thus, as shown in Figure 1b, a clock sorting module is designed to sort the clocks. The rising edges and the falling edges will be captured both, and their values will be used to calculate the fine timestamp in the decode module.

The other modules of the NUMP TDC will be introduced with the temperature correction module later.

### 2.2. Temperature Characteristics of Delay Chain

In previous work, Pan et al. [18] used a complementary metal oxide semiconductor (CMOS) inverter as an example to qualitatively analyze the effect of temperature on the delay chain of Cyclone II FPGA devices and suggested that carrier mobility and threshold voltage affected the propagation delay. A series of experiments were performed, and results indicated that the temperature fluctuations had similar effects on all bins in a delay chain with a limited length (less than 105 delay units). Although Cyclone 10 LP FPGA is a newer product in the Cyclone series FPGA, its positioning and underlying logical architecture are similar to the Cyclone II FPGA. The logic array block (LAB) consists of logic elements (LEs) and has the same carry chain structure [29,30]. Therefore, their delay chains have similar temperature characteristics. The detail about the delay chain has been discussed further in Section 5.

The principle of the NUMP TDC is quite different from a wave-union TDC [15]. Therefore, the conclusion reached in the previous study could not be directly used to support the temperature correction method for this paper. Based on Pan’s research, a further conjecture could be proposed: since the variation in propagation delay of each delay unit (hereinafter called a ‘cell’) is roughly the same when the temperature changes, if the input of a delay chain is a clock signal instead of a hit signal, the delay at the delay chain’s end cell is always longer than at the delay chain’s front end cell, since the delay effect gradually accumulates in each cell. As the number of cells increases, it is obvious that the propagation delay of the clock signals will increase linearly. 

**Figure 2:**
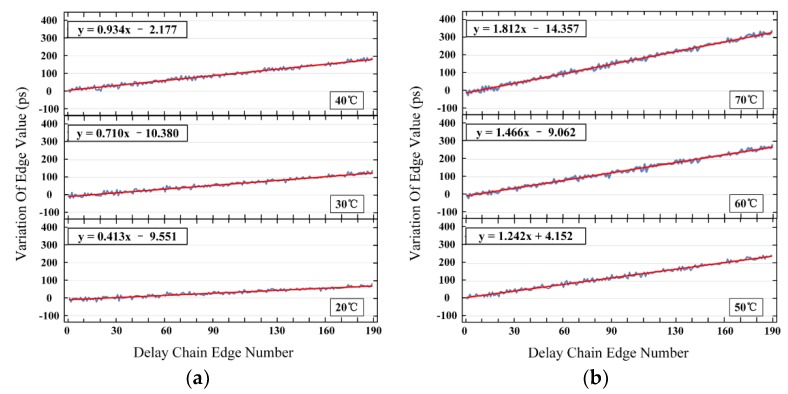
Variation of edge values at different temperatures compared to the clock sorting operation results at 10 °C. The horizontal axis shows the edge number, and the vertical axis is the variation of edge value. The blue lines and red lines represent the true curves and fitted curves, respectively. There are 95 cells in each sub-delay chain, and the first sub-delay chain’s variation of edge values are shown here. (**a**) The changes of the propagation delays at 20 °C , 30 °C and 40 °C; (**b**) The changes of the propagation delays at 50 °C , 60 °C and 70 °C.

Experimental studies were performed to validate this hypothesis. The clock sorting operation was performed repeatedly, with the temperature changed from 10 °C to 70 °C at 10 °C per step. The results of the clock sorting operation measured at 10 °C were selected as the references. The measurements at other temperatures were subtracted from the references to obtain the changes of the propagation delays (hereinafter called the ‘variation of edge values’) for each cell. Figure 2 shows the variation of the edge values measured at temperatures ranged from 20 °C to 70 °C at 10 °C per step.

As shown in Figure 2, the edge values of each cell for FPGA at different temperatures were different from the edge values obtained by the clock sorting operation at 10 °C. Therefore, the temperature of the FPGA (more precisely, the delay chain) could be measured using the variation in edge values.

**Figure 3:**
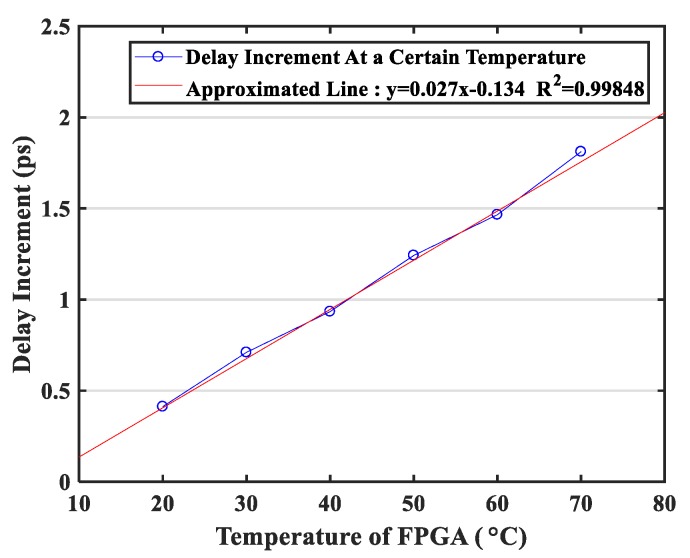
Variation in delay increment with a field-programmable gate array (FPGA) temperature.

Figure 3 shows the variation in delay increment with FPGA temperature, which was the slope of the change in Figure 2. This graph had excellent linearity with an R^2^ value that reached 0.998. The slope of the approximated line was 0.027, meaning that for each increase (or decrease) temperature by 1 °C, the propagation delay variation would increase (or decrease) by 0.054 ps (0.027 × 2). Although this linear relationship existed between the propagation delay and the temperature, this relationship was not a prerequisite for the temperature correction method proposed in this paper.

### 2.3. Temperature Measurement Structure

The basic concept of the proposed temperature correction method for the NUMP TDC is as follows. A code density test is used to test the edge values of one or more cell(s) in the delay chain, and the results are compared with the edge values stored in the LUT. If the edge value variation exceeds a specified temperature correction threshold, the NUMP TDC switches to temperature correction mode, and the original LUT is updated to consider the current temperature state.

**Figure 4:**
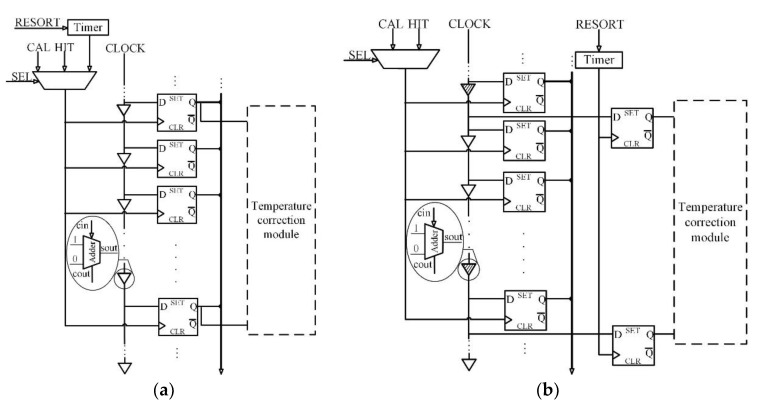
Two methods for temperature measurement. (**a**) Monitoring through dedicated registers. (**b**) Monitoring through the thermometric registers. The shadowed cells in Figure 4b are the thermometric cells; the only difference between these cells and normal cells is that the thermometric cells are extra paired up with thermometric registers.

The FPGA delay chain temperatures could be measured using two methods, as shown in Figure 4.

For the first method, the temperature states are monitored using dedicated registers, as shown in Figure 4a. The advantage of this method is that the results accurately reflect the temperature states of the delay chain. However, this method has an obvious shortcoming: the delay chain states cannot sample simultaneously by the hit signal and the resort signal, so a timer is required to periodically test the temperature. This inevitably results in a larger dead time.

The second method is shown in Figure 4b and uses thermometric registers besides the delay chain to monitor variations in the edge values of the thermometric cells. This allows the temperature state of the delay chain to be monitored at any time. The biggest advantage of this method is that it is independent of time measurement and temperature measurement. The thermometric registers are not as precise as the dedicated registers and may not be as accurate at capturing the delay chain temperature. However, this difference can be minimized by using a logic lock tool in the design platform. By deploying the thermometric registers around the delay chain, the clock states in the delay chain can be almost simultaneously fed into two types of registers. This makes it possible to use additional thermometric registers rather than dedicated delay chain registers to monitor the delay chain temperature. Thus, for our proposed temperature correction module, the second method was utilized.

### 2.4. Top-Level Diagram of NUMP TDC with Temperature Correction Module

**Figure 5:**
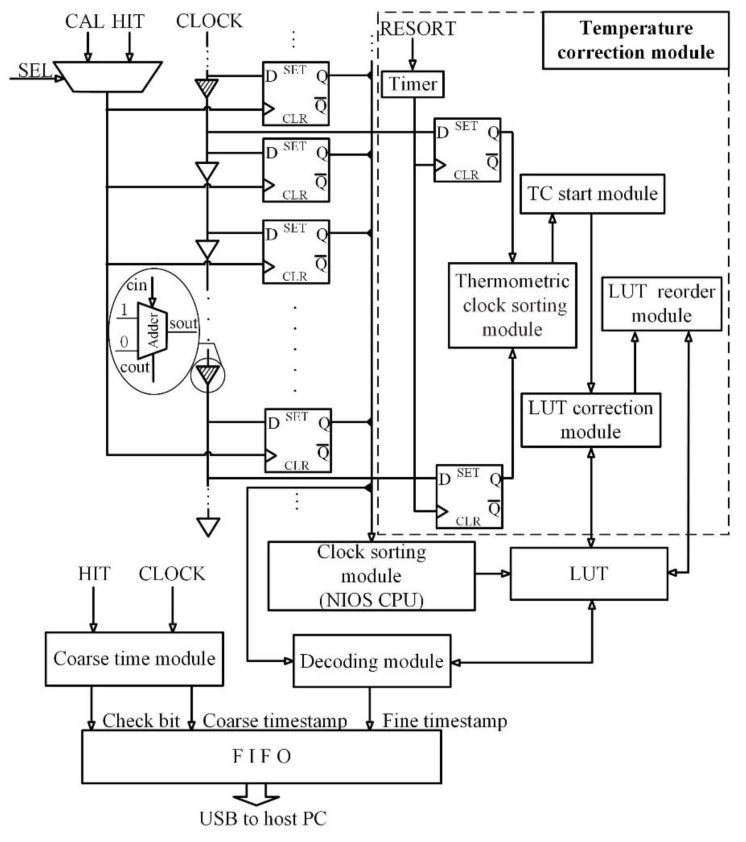
Diagram of NUMP TDC with the temperature correction module. For more clarity, this diagram only describes one channel TDC and omits some of the details already expressed earlier.

Figure 5 provides a diagram of the NUMP TDC with the temperature correction module. This diagram has two major differences from a conventional NUMP TDC. Firstly, two additional thermometric registers are deployed at both ends of the delay chain to monitor variations in the edge values. Secondly, the LUT is updated in real-time to compensate for temperature effects on the delay chain.

The NUMP TDC with the temperature correction module has three operation modes. In the initialization clock sorting mode, a calibration signal CAL is selected to latch the clock states in the registers, and the sampling results are calibrated by the clock sorting module, which is based on the internal netware input/output subsystem (NIOS) CPU of the FPGA. The phases (the edge values) of each cell are calibrated, sorted, and stored in the LUT. Once this step is complete, the time intervals can be measured by the NUMP TDC.

In normal operation mode, clock states with random phase shifts are sampled simultaneously by the hit signal. The sampled states of the clocks are sent to the decoding module to calculate the fine timestamp. The hit signal is also fed to the coarse time module, which outputs a 16-bit course timestamp and a check bit to correct the metastable error. The fine timestamp and the coarse timestamp with its check bit are then combined and buffered in the first input first output (FIFO) buffer and transmitted to the host PC by USB cable. Simultaneously, the thermometric clock sorting module periodically measures the edge values of the thermometric cells. The variation in edge values is fed to the temperature correction start module (hereinafter called the ‘TC start module’) for analysis. The NUMP TDC will automatically switch to temperature correction mode if the variation is larger than a given threshold.

In the temperature correction mode, the edge values are corrected. The edge values of each cell are calibrated and sorted, and the LUT is updated. Once the process is completed, the TDC switches back to the normal operation mode.

## 3. Implementation 

### 3.1. Top-Level Diagram of Temperature Correction Module

**Figure 6:**
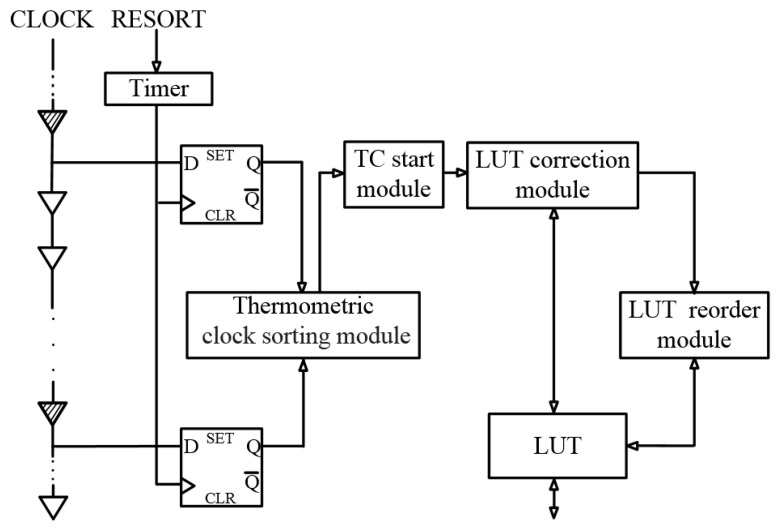
Diagram of the temperature correction module.

A diagram of the temperature correction module is shown in Figure 6.

Since temperature variations tend to occur very slowly, it is not necessary to continuously monitor the temperature. The timer is used for periodic measurement of the delay chain temperatures using the thermometric clock sorting module. After each measurement, the absolute value of the difference between the edge values of the thermometric cells is fed to the TC start module for analysis of the current temperatures. The analysis process is as follows. If there is no temperature change trend identified, the TDC will remain in normal operation mode. If a temperature change trend is identified, the module will check if the change is larger than the given threshold. If so, the TDC switches to temperature correction mode, i.e., temperature correction will be launched, and the time measurement process will be suspended. Otherwise, the TDC will remain in normal operation mode.

Once the temperature correction starts, the edge values in LUT will be individually corrected by the LUT correction module. This disturbs the original order of the edge values. Therefore, a LUT reorder module is introduced to reorder the edge values in the LUT after each correction. Once the reorder process is completed, and the entire temperature correction process is finished, the TDC switches back to the normal operation mode.

The temperature correction module is performed in real-time and online.

### 3.2. Clock Sorting Operation for Temperature Measurement

This paper proposed a simple method to monitor the temperature of delay chains. Two thermometric registers are deployed at both ends of the delay chains, and a thermometric clock sorting operation is carried out periodically to obtain the edge values of the thermometric cells. The variation in edge values is calculated and fed into the TC start module to determine whether temperature correction should be performed.

The thermometric clock sorting operation is based on the code density test [13]. Two signals are used during this process—the reference signal C_0_ and the signal to be measured C_X_—which is the thermometric cell clock. The frequency of the resort signal is 6.7821 MHz, which is not related to the system clock, which is 400 MHz. Thus, the probability for each cell to be sampled is the same. A large number of random samples are performed to evenly distribute the resort signals at various positions across the whole clock cycle. The proportion of measurements is calculated between C_0_ and C_X_ for each of the four types “10”, “11”, “01”, and “00”. The results are multiplied by the clock cycle to obtain the edge values of C_X_. 

**Figure 7:**
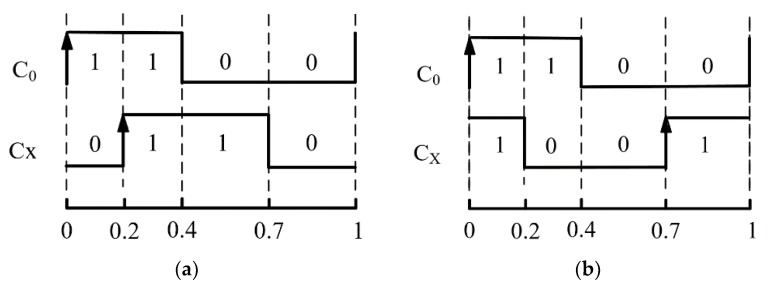
The phase relationship between the reference signal C_0_ and the signal being measured C_X_: (**a**) Rising edge of C_X_ arrives ahead of its falling edge for a clock cycle of C_0_; (**b**) Falling edge of C_X_ arrives ahead of its falling edge for a clock cycle of C_0_.

There are always two possible relationships between C_0_ and C_X_. If the ratio between the four cases is 0.2, 0.2, 0.3, and 0.3, the rising and falling edge positions will be restricted to the two cases shown in Figure 7.

For the initialization clock sorting operation, CAL_1 is used to obtain two possible relationships between the reference clock C_0_ and any other clock C_X_. The role of CAL_2 is to distinguish between these two cases and is not required in temperature correction mode. By transmitting the results of clock sorting operation to the host computer for analysis, the phase relationship between each thermometric cell’s signal C_X_ and the reference signal C_0_ can be confirmed clearly.

**Figure 8:**
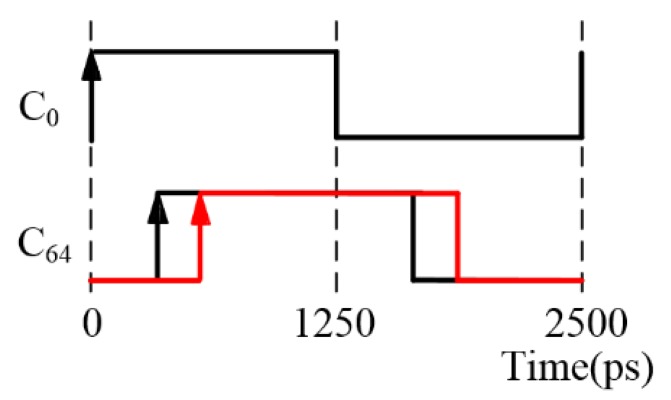
Variation of edge for the 64th cell as the temperature changes. The black line represents the original edge value at 10 °C, and the red line represents the edge value after the temperature increases to 70 °C.

It should be noted that the thermometric cells at both ends of the delay chains referred to in this paper do not represent the first and last cells in the absolute sense, but rather the cells at the start and end positions of the delay chain. In order to save computing resources and time of the whole correction process, the cell with a number that is a power of 2 can be considered as the thermometric cell in priority. For example, the 64th cell can be selected if there are 95 cells in each delay chain. Figure 8 shows the clock signal of the 64th cell at TDC temperatures of 10 °C and 70 °C. As the temperature increases from 10 °C and 70 °C, the value of the rising edge changes from 363 ps to 574 ps, i.e., a 211 ps edge increment occurs, which accounts for a delay of 8.44% of the total clock cycle of 2500 ps.

This variation in edge values due to changes in temperature is small relative to the overall clock cycle. Therefore, in contrast with the thermometric clock sorting operation module, it is not necessary to count the proportions of each of the four cases, as counting a single case will be sufficient. This greatly reduces programming complexity and saves FPGA resources. For example, to calculate the variation in the rising edge of the 64th cell, we only need to count the frequency of “10” cases. The frequency of each case is divided by the total number and multiplied by the system clock cycle to get the edge values at the current temperature. After the initialization clock sorting operation is completed, the thermometric clock sorting operation starts after one timer period. For any specific temperature, the variation in edge value can be represented by Equation (1) as:

(1) $$\Delta edge=edge_{n}^{*}-edge_{n}$$

where *n* is the thermometric cell number, $edge_{n}^{*}$ is the rising/falling edge value when the temperature varies, and $edge_{n}$ is the original rising/falling edge value.

A positive value of Δedge indicates that the current temperature of the delay chain is higher than the original temperature and the temperature rise mark bit is asserted. A negative value of Δedge indicates that the current temperature of the delay chain is lower than the original temperature and the temperature drop mark bit is asserted. Both Δedge and the mark bit are sent to the TC start module to determine the temperature trend.

### 3.3. Temperature Correction Start Module

The TC start module operates as follows. It firstly assesses whether the current temperature of the delay chain has increased, decreased, or is substantially unchanged compared to the previous temperature. If there is a clear and strong temperature trend, temperature correction is launched. Otherwise, TDC remains in normal operation mode.

For accurate temperature trend measurement, the temperature measurement frequency, which is controlled by a timer, should be set to a suitable frequency. However, a temperature measurement frequency that is too high will increase the randomness of the temperature measurement, which may increase the probability of incorrectly identifying a temperature trend. Therefore, strict trend judgment criteria are required. In this paper, two counters, named *rise_cnt* and *fall_cnt*, were used to determine the temperature trend. Both counters started at zero and were incremented each time its corresponding temperature mark bit was asserted. The difference and sum of *rise_cnt* and *fall_cnt* were both calculated. Before the sum of the two counters reached a pre-defined value, if the difference between the two counters was greater than half this value, a temperature trend was identified with the larger counter, indicating whether the temperature was rising or falling.

Once it is evident that there is a temperature trend, a threshold judgment is performed. If Equation (2) is true, the temperature correction process is launched, and the LUT correction module and the LUT reorder module are sequentially triggered to correct and sort edge values and store the new values in the LUT.

(2) $$\left|\Delta edge_{tail}-\Delta edge_{van}\right|>threshold$$

where $\Delta edge_{tail}$ and $\Delta edge_{van}$ are the variations of the edge values of the thermometric cells matched with the thermometric registers, and threshold is a pre-defined temperature correction threshold.

Note that once temperature correction is launched, the LUT used for the decoding process must be updated, so time measurement cannot be performed during the temperature correction process and must wait until it has been completed. The introduction of a *threshold* prevents the TDC from performing unnecessary temperature corrections for temperature fluctuations within a small range. The *threshold* is a parameter that should be determined by executing the clock sorting operation repeatedly while the ambient temperature varies and can be set according to the FPGA temperature adaptability and the working environment. 

### 3.4. LUT Correction Module

Since the delay chain has a cumulative effect on the delay of the input clock signal, the delay effect of temperature on the delay chain also has a cumulative effect, i.e., cells at the back of the delay chain will occur larger edge value variation than the cells at the front of the delay chain. The main task of the LUT correction module is to correct the edge values according to the position of the cells in the delay chain.

For example, for the first sub-delay chain, the correction formula will be as follows:

(3) $$edge_{i}^{*}=edge_{i}\pm \;\frac{threshold}{num_{tail}-num_{van}}\times num_{n}$$

where $num_{n}$ is the number of the *n*th cell of the delay chain, which is equal to *n*, $num_{van}$ and $num_{tail}$ are the numbers of the thermometric cells, $edge_{i}$ is the current edge value, and $edge_{i}^{*}$ is the edge value after correction. Each cell has a rising and falling edge value, so there will be twice as many edge values as cells, i.e., *i* is equal to 2*n* or 2*n* − 1. The effect of temperature on the delay chain considers each cell as a minimum element, so the same correction parameters are used to correct both types of edges for the same cell. The other three sub-delay chains have a similar correction process to the first sub-delay chain. Each cell is corrected using the same correction parameters for that cell position, e.g., the edge values of the last cell of the second sub-delay chain are corrected using the same correction parameters as the last cell of the first sub-delay chain.

Because of the cross-clock cycle phenomenon, $edge_{i}^{*}$ need to be checked after corrected by Formula (3). If $edge_{i}^{*}$ is larger than the system clock cycle, the real $edge_{i}^{*}$ is equal to its value less than the system clock cycle. If $edge_{i}^{*}$ is smaller than 0, the real $edge_{i}^{*}$ is equal to its value plus the system clock cycle. The cross-clock cycle phenomenon has been discussed further in Section 3.5.

In addition, to avoid repeatedly triggering temperature corrections at the same temperature, the parameter $edge_{n}$ in Formula (1) should be updated by reading the corrected LUT after each LUT correction.

### 3.5. LUT Reorder Module

**Figure 9:**
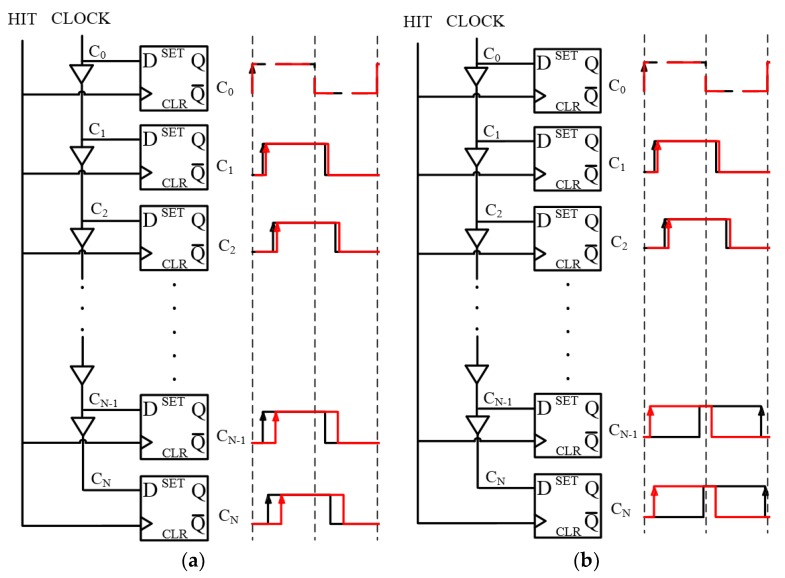
Schematic diagram of the edge values of cells when the temperature rises. The black line represents the original edge value, and the red line represents the edge value when the temperature rises. (**a**) Edge values increase but do not cross clock cycles; (**b**) Edge values increase and cross clock cycles.

After executing the initialization clock sorting operation and obtaining the edge values of the clock signal corresponding to each cell, the edge values are sorted and stored for each cell number in LUT. Once the length of the delay chain reaches a certain value, the delay to the input clock signal will be large enough to result in a cross-clock cycle phenomenon. Therefore, there may be multiple cells with similar phases in the delay chain, as shown in Figure 9. 

As shown in Figure 9a, in its original state, the rising edge value of C_N_ is slightly smaller than C_2_. Therefore, the rising edge value of C_N_ must be placed before the rising edge value of C_2_ during the sorting process, before being stored in the LUT. When the temperature rises, the edge increment of the delay chain’s back end cells must be larger than the edge increment of the front end cells due to the cumulative effect of the delay. Therefore, after performing edge correction, the rising edge value of C_N_ will be larger than the rising edge value of C_2_. In contrast, as shown in Figure 9b, if the edge value is very close to the clock cycle, then as the temperature rises, the cross-clock cycle phenomenon will occur. (Note the situation when the temperature drop is similar but has not been described here). As a result, the order of the edge values in the original LUT will be disturbed. Therefore, the LUT reorder module should be introduced.

The FPGA sort operation is more complicated than a software sort, as the use of arrays requires a high logic resource consumption. Additionally, the design platform may get trapped in the analysis and synthesis process due to the use of the large array and the loop structure. 

Therefore, pairwise comparison is adopted to construct the reorder module. Each time two edge values are read from the LUT, the two adjacent edge values are compared, and the larger value is retained. Once the pairwise comparison is completed, the larger value is saved. The new and the old larger values are further compared, and the larger value is retained as the alternative to the biggest edge value for the current round of comparison. The alternative biggest edge value is constantly updated for each pairwise comparison. When all the edge values in LUT have been compared, the alternative biggest edge value (the biggest edge value in the current round of comparison) is placed in the storage address of the last read data value. In order to prevent data loss, the data originally at this address is stored in the address of the biggest edge value. In summary, each round of comparison will identify the biggest edge value for that round and store that value and its cell number at the end of the LUT. The edge value and cell number, which were previously stored at this position, will be stored in the original address of the biggest edge value.

In order to illustrate this process clearly, information on the first 10 edges was extracted from a real LUT so that the comparison process could be explained in detail. The edge value units were picosecond.

The first round of comparison is shown in Table 1. The cell number 346 and its edge value 92 were retained in the 10th memory address. The edge values stored in the first 9 memory addresses would be sorted by the second round of comparison. The updated LUT for the second round of comparison is shown in Table 2.

For the second round of comparison, the even memory address (10th) data was missing for the fifth data comparison. Therefore, the edge value of the last even memory address (8th) was read instead and compared with the odd memory address (9th). After the second round of comparison, the cell number 259 and its edge value 53, which were stored in the 4th memory address, were used to replace the cell number 291 and its edge value 36 stored in the 9th memory address. The third round of sorting is shown in Table 3, which would sort the edge values stored in the first 8 memory addresses. 

Based on this process, the sorting process will not be repeated for each memory location. If there are *m* edge values, then *m* − 1 rounds of comparison are required. Once the reorder process is completed, the overall temperature correction process is finished, and the TDC switches back to the normal operation mode.

## 4. Experimental Validation

A two-channel NUMP TDC was implemented in a 60 nm Altera Cyclone 10 LP FPGA (10CL120YF780C8G) to validate the proposed temperature correction method. Each channel contained 380 cells to generate 380 delayed 400 MHz clocks.

### 4.1. Variation of Edge Values with Temperature Changes

**Figure 10:**
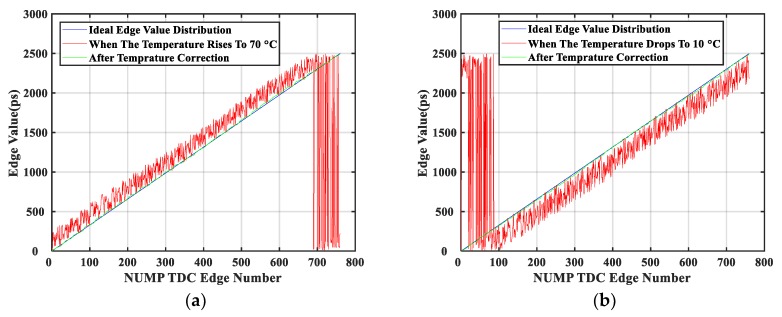
Distribution of corrected and uncorrected edge values when the temperature varies: (**a**) Temperature increases to 70 °C; (**b**) Temperature drops to 10 °C.

The initialization clock sorting operation was executed at 40 °C. As the temperature varied, Figure 10 shows the 760 edge values in the LUT with and without the temperature correction module. The ideal edge value distribution was also shown in these graphs for ease of comparison. 

For discussion purposes, Figure 10a is taken as an example, which showed the effect of temperature rises. The red line in this figure showed that the edge values at the front had larger variations than the edge values at the back. The reason for this was that although the edge values at the front were small, there were a large number of cells match them. Due to the cumulative delay effect, the latter part of the delay chain was more affected by temperature than the front part of the delay chain. This was reflected in the edge values by a higher change in variation. However, the edge values of the cells at the end of the delay chain spanned a whole system clock period due to the rise of temperature, so its edge values were restarted from zero. Therefore, the temperature correction module was required. After LUT correction and reordering was completed, the distribution of the edge values in the LUT was shown by the green line in Figure 10. The offside edges had disappeared, and the LUT could be reused for decoding to calculate the fine timestamp. Figure 10b shows the effect of temperature decreases, which was similar to the effect of temperature rises, and is not described here to avoid repetition.

### 4.2. Nonlinearities

TDC nonlinearities would directly affect the resolution of the system. The most commonly-used metrics to characterize TDC nonlinearities are differential nonlinearity (DNL) and integral nonlinearity (INL) [31,32]. DNL describes the difference between each bin width and the ideal bin width, which is also known as the least significant bit (LSB) and reflects the degree of nonlinearity of single bin width. INL represents the cumulative offset, and its value can be obtained by integrating the DNL values for some conventional TDC.

However, the edges of all the time bins of the NUMP TDC were measured independently. Thus, the DNL errors of the NUMP TDC did not accumulate to produce INL errors like conventional TDCs. In the decoding stage, using the binary search algorithm and the sampled delayed clock states latched by hit signal, the fine timestamp region could be narrowed constantly and located finally when searching two adjacent memory addresses. The mean of the corresponding edge values was calculated as the fine timestamp. Note that the edge values used in the decoding process were confirmed accurately by the initialization clock sorting operation. In conclusion, due to the introduction of the clock sorting module, the bin-by-bin calibration was executed to avoid the negative influence of INL that might be caused by uneven bin sizes. Edge values were sorted and stored in the LUT during the initialization clock sorting mode. In normal operation mode, each fine timestamp measurement result only depended on the two adjacent edge values obtained at the final stage of decoding but was not related to the other edge values in the delay chain. Thus, the NUMP TDC had only DNL because the last bin obtained by the decoding module might be not equal to the LSB and had no INL.

**Figure 11:**
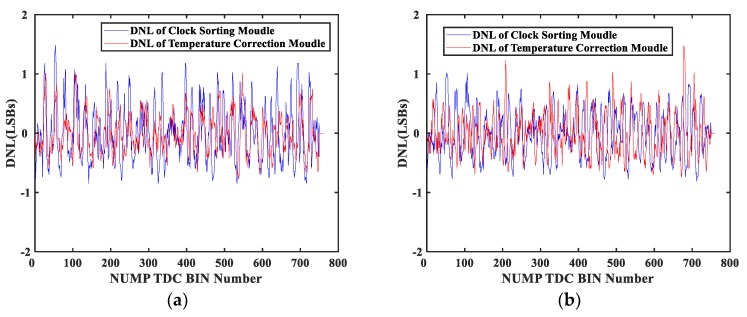
DNL (differential nonlinearity) of the NUMP TDC: (**a**) Temperature rises from 40 °C to 70 °C; (**b**) Temperature drops from 40 °C to 10 °C.

The DNL of the delay chain when the temperature changed from 40 °C to 70 °C and 10 °C are shown in Figure 11a,b, respectively. 

When the temperature rose from 40 °C to 70 °C, and after the temperature correction, the DNL was within [−0.81, 1.20] of LSB. The RMS of the DNL was 0.34 ps. In contrast, the DNL of the initialization clock sorting operation was tested, and the DNL was within [−0.84, 1.48] of LSB. The RMS of the DNL was 0.48 ps. When the temperature dropped from 40 °C to 10 °C, and after the temperature correction, the DNL was within [−0.74, 1.48] of LSB. The RMS of the DNL was 0.29 ps. For the initialization clock sorting operation at 10 °C, the DNL was within [−0.81, 1.01] of LSB. The RMS of the DNL was 0.42 ps. The DNL obtained by the temperature correction module provided a similar level of accuracy as the DNL values obtained by the initialization clock sorting operation, which indicated that the LUT updated by the temperature correction module was practical.

### 4.3. TDC Measurement

**Figure 12:**
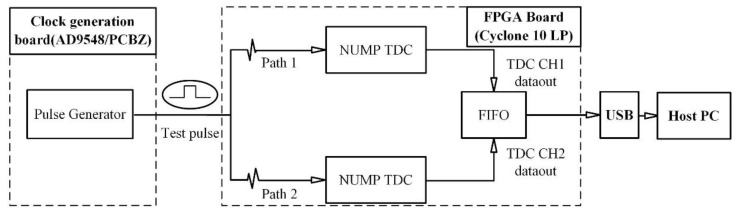
The diagram of TDC measurement. The external test pulses were sent to the FPGA through a low-voltage differential signaling (LVDS) port and fed to the two TDCs.

As shown in Figure 12, the external pulses generated by an Analog Devices Inc. clock generation board (AD9548/PCBZ) were sent to the FPGA through a low-voltage differential signaling (LVDS) port and fed into the two TDCs. The differences between the two TDC measurements were calculated in the FPGA and transmitted to the PC through a USB cable.

**Figure 13:**
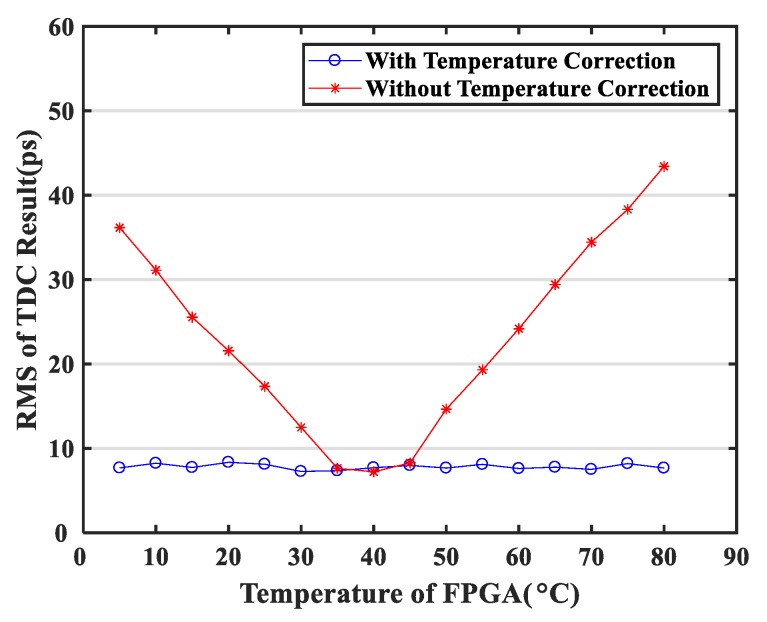
Single-shot resolutions of TDC for temperature variations from 5 °C to 80 °C. The initialization clock sorting operation was executed at 40 °C.

The performances of the two TDCs were characterized by calculating the RMS of the differences between the two TDC measurements. As shown in Figure 13, the TDC resolution reduced significantly due to temperature changes. However, the proposed temperature correction method successfully eliminated the influence of temperature in the range of 5 °C to 80 °C on the TDC resolution. 

**Figure 14:**
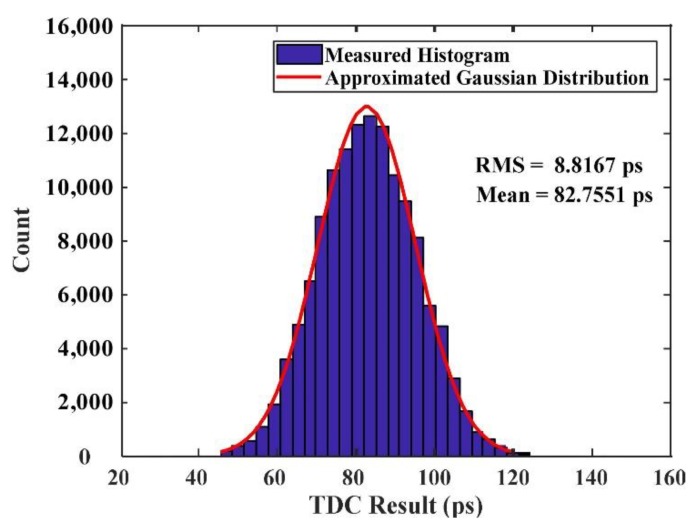
Distribution of the differences between the two TDC measurements in the temperature range from 5 °C to 80 °C. Automatic temperature corrections were applied, and the overall TDC resolution was 8.8 ps.

Figure 14 shows a statistic histogram of more than 100,000 measurement results collected during this experiment at different temperatures. This histogram clearly showed that although the temperature changed, the TDC measurement results still showed an excellent Gaussian distribution. The overall TDC resolution was 8.8 ps (RMS), which contained all the error factors, including DNL and jitter. Note that all RMS values given in this paper represented the single-shot resolution, which was 1/$\sqrt{2}$ of the dual-channel resolution.

## 5. Discussion

### 5.1. Correction Parameters of Temperature Correction Module

In our design, four sub-delay chains of the same channel were corrected simultaneously. This method had a potential problem: the four sub-delay chains of the same channel might not show similar delay characteristics for temperature changes. In other words, when a phase shift occurred in one sub-delay chain, which was sufficient to trigger a temperature correction, it was not clear whether the other three sub-delay chains would change to the same extent. 

To examine this issue further, the linear fitting results of delay chain_1 to delay chain_4 are listed in Table 4, which were similar to the equations shown in Figure 2. Furthermore, based on the equations shown in Table 4, the linear fitting results of slopes are shown in Table 5, which were similar to the equations shown in Figure 3.

As shown in Table 4, the four sub-delay chains had similar delay characteristics, and the slopes of the fitted functions were only slightly different. ΔK_MAX_ in Table 4 was used to set Delay chain_1 as the reference. Although the intercepts were quite different, the correction process only used the correction parameters related to each cell number and added the original edge values. Thus, the correction effect was not related to the intercepts. As shown in Table 4, the maximum difference in slope was 0.032 when the temperature varied from 10 °C to 70 °C. At 70 °C, the maximum correction error was 0.032 × 95 = 3.04 ps, which was smaller than the LSB (3.2895 ps).

As shown in Table 5, the slope of the fitted equations varied between 0.027 to 0.028, which further indicated that the four sub-delay chains had similar delay characteristics across a wide temperature range. Each cell’s delay would increase (or decrease) by 0.054 ps to 0.056 ps when the temperature rose (or drops) by one degree Celsius.

### 5.2. Carry Chain Differences between Cyclone V and Cyclone 10 LP

The NUMP TDC that we proposed was based on the Altera 28 nm Cyclone V FPGA (5CEBA4F23C7N) and implemented with 400 delayed 500 MHz clocks, where each sub-delay chain had 100 cells. NUMP temperature stability was also tested. The NUMP TDC based on the Cyclone V FPGA was not sensitive to temperature fluctuations. The resolution of the TDC without temperature correction was only slightly different and changed from 5.4 ps to 7.3 ps when the temperature was increased from 20 °C to 56 °C [28]. In contrast, the NUMP TDC based on the Cyclone 10 LP FPGA was very sensitive to temperature fluctuations. As could be seen from the experimental results in Figure 12, the resolution of the NUMP TDC without temperature correction was dramatically changed from 7.2 ps to 43.4 ps when the temperature increased from 40 °C to 80 °C.

**Figure 15:**
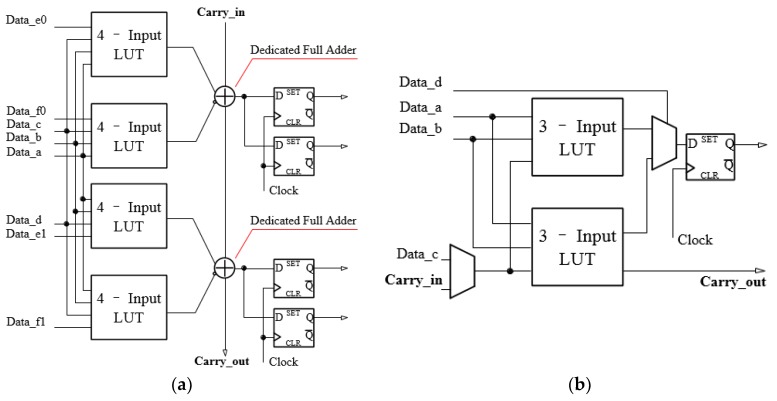
Comparison of the carry chain: (**a**) carry chain in Cyclone V FPGA; (**b**) carry chain in Cyclone 10 LP FPGA.

The 60 nm Cyclone 10 LP FPGAs are the new generation of 28 nm Cyclone V FPGAs due to their unique advantages, including low power consumption and low cost. These FPGAs also differ in terms of their underlying logical resources [29,33]. Each logic array block (LAB) of a Cyclone V FPGA consists of 10 adaptive logic blocks (ALMs), whereas a Cyclone 10 LP FPGA has 16 logical elements (LEs). In the TDC field, the main difference between the two FPGAs is reflected in the carry chain processing, which is a constituent part of the delay chain. For the Cyclone V FPGA, the carry chain consists of “dedicated full adders”. However, in Cyclone 10 LP FPGA, the carry chain is formed by the LUT. Figure 15 shows these two distinct carry chain processing methods.

It was evident that the Cyclone V FPGA and Cyclone 10 LP FPGA had different carry chain structures. Although the difference was not obvious in terms of the propagation delay, there were huge temperature stability differences, which might result in a large difference in temperature characteristics for the same TDC scheme implemented on different devices.

### 5.3. Resource Usage and Power Consumption

The resources used in NUMP TDC with temperature correction majorly included logic cells, logic registers memory bits, phase-locked loops (PLLs), and routing resources. The logic utilizations of NIOS CPU, NUMP TDC, and temperature correction module are compared in Table 6. Note that multiple TDC channels could share one temperature correction module, and one NIOS CPU could support all the TDC channels in the FPGA. Theoretically, 40 to 70 channels of NUMP TDCs could be implemented with temperature correction in a single Cyclone 10 LP FPGA. 

The consumption of a TDC was determined by both the resource usages and the event rate. The power consumption of the NUMP TDC system worked at an event rate of 1 MHz was calculated using the Cyclone 10 LP Early Power Estimator. The results showed the delay chains operated with a clock frequency of 400 MHz dominated the power consumption of the NUMP TDC (39 mW). The power consumptions of the NIOS CPU, a single channel of NUMP TDC, and the temperature correction module were 58 mW, 40 mW, and 1 mW, respectively. 

### 5.4. Features of the Temperature Correction Method

The temperature correction method proposed in this paper has some unique features compared to many of the conventional temperature correction methods. 

**Figure 16:**
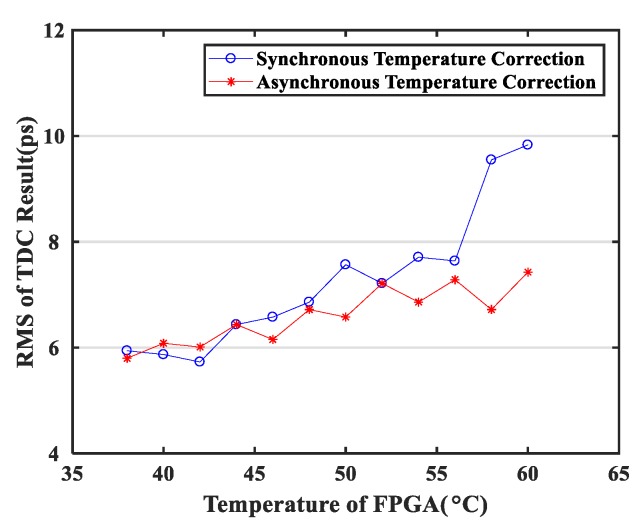
Test results of TDC at the same temperatures for both synchronous and asynchronous temperature correction.

Firstly, it supports asynchronous temperature correction between multiple channels. Although the overall FPGA chip will have the same ambient temperature, the temperature states of the individual channels may not be identical, so the temperature characteristics of the individual delay chains may be different. Therefore, whether the temperature is measured by an external temperature sensor [16,17] or a dedicated delay chain [18], any temperature correction will be triggered for all delay chains, which will inevitably introduce a certain level of error. The temperature correction method proposed in this paper could measure the temperature state of each channel’s delay chain and determine whether each channel needs to be corrected, thus providing individualized delay chain correction for each channel. Figure 16 compares the effect of synchronous correction and asynchronous correction through an internal signal test.

Secondly, there is no requirement for intensive experiments at an early stage to determine the relationship between the temperature and correction parameters, which is convenient for reproducibility with different FPGA boards. For different production batches using the same series of FPGA, the delay characteristics and temperature characteristics of the delay chain may not be identical due to slight variations in the manufacturing process. The difference in delay characteristics can be fully calibrated during the initialization clock sorting operation. For the temperature sensitivity, to explore the corresponding relationship between the correction parameters and temperature, for other temperature correction methods, a special temperature control device is required to calibrate the correction parameters for multiple temperature states. This process is cumbersome, and the correction parameters cannot be generalized, i.e., the exact same experiments need to be repeated for each FPGA board [16,17,18]. However, these experiments are not necessarily using the temperature correction method proposed in this paper. Although the temperature characteristics of different FPGAs are not linear, as shown in Figure 3, the cumulative characteristics of the delay chain for the clock signal input is still constant, as shown in Figure 2. 

Thirdly, there is no requirement for an external component, such as a temperature sensor. The method proposed in this paper accurately measures the temperature measurement state of the delay chain by deploying dedicated thermometric registers at both ends of the delay chain. This greatly reduces the error introduced by temperature measurement.

Finally, the method proposed in this paper could offer excellent flexibility. While deploying more thermometric registers can improve the accuracy of the temperature measurement, this can also be achieved by obtaining the mean of the edge value variation. The strategy of using only two thermometric registers at both ends of a single sub-delay chain is more practical and can save resources while still achieving a good temperature correction effect. Additionally, a larger threshold can be set to reduce the frequency of correction and dead time. For applications that are not sensitive to the dead time, a smaller threshold can also be used to increase the temperature stability of the TDC.

Most FPGA devices have logic cells close to each other with very similar performances. Thus, it is expected that there are no large thermal gradients along the delay line. Therefore, the temperature correction method presented in this paper is probably applicable to other technologies/devices, although it was only validated in Cyclone 10 LP FPGA in this study.

## 6. Conclusions

In order to achieve high performance within a wide temperature range, this paper proposed a temperature correction scheme that integrated a temperature monitor and automatic correction for NUMP TDC. The effect of temperature on the delay chain had been discussed, and a method to monitor the temperature state of the delay chain by measuring changes in the edge value of the thermometric cells had been proposed. When the variation in the edge value exceeded a given threshold, temperature correction was launched. The temperature correction was performed in the FPGA in real-time.

In conclusion, this paper developed a low-cost and high-performance method that could effectively reduce the effect of temperature fluctuations on an FPGA-based NUMP TDC. Using this method, a resolution of 8.8 ps RMS over a wide temperature range from 5 °C to 80 °C had been achieved in a NUMP-TDC implemented in a Cyclone 10 LP FPGA.

## Figures and Tables

**Table 1:** First round comparison.

Memory Address	Cell Number	Edge Value (Uncorrected)	Edge Value (Corrected)	The Bigger Edge Value	Alternative	Alternative Address
1	1	0	0	46	46	2
2	144	3	46			
3	202	5	22	53	53	4
4	259	7	53			
5	143	8	52	52	53	4
6	26	10	45			
7	201	20	41	41	53	4
8	11	21	33			
9	291	26	36	92	92	10
10	346	34	92			

**Table 2:** Second round comparison.

Memory Address	Cell Number	Edge Value (Uncorrected)	Edge Value (Corrected)	The Bigger Edge Value	Alternative	Alternative’s Address
1	1	0	0	46	46	2
2	144	3	46			
3	202	5	22	53	53	4
4	259	7	53			
5	143	8	52	52	53	4
6	26	10	45			
7	201	20	41	41	53	4
8	11	21	33			
9	291	26	36	36	53	4
10	346	—	—	92	—	—

**Table 3:** Third round comparison.

Memory Address	Cell Number	Edge Value (Uncorrected)	Edge Value (Corrected)	The Bigger Edge Value	Alternative	Alternative’s Address
1	1	0	0	46	46	2
2	144	3	46			
3	202	5	22	36	36	4
4	291	7	53			
5	143	8	52	52	52	5
6	26	10	45			
7	201	20	41	41	52	5
8	11	21	33			
9	259	—	—	53	—	—
10	346	—	—	92	—	—

**Table 4:** Fitting results of edge values’ variation.

Temperature	Delay Chain_1	Delay Chain_2	Delay Chain_3	Delay Chain_4	ΔK_MAX_
20 °C–10 °C	y = 0.412x − 9.551	y = 0.416x − 10.838	y = 0.416x − 11.524	y = 0.398x − 11.503	0.014
30 °C–10 °C	y = 0.710x − 10.38	y = 0.727x − 14.268	y = 0.732x − 16.601	y = 0.709x − 16.551	0.022
40 °C–10 °C	y = 0.934x + 2.176	y = 0.942x − 0.444	y = 0.954x − 3.858	y = 0.923x − 3.355	0.020
50 °C–10 °C	y = 1.242x + 4.152	y = 1.253x + 0.278	y = 1.242x + 4.152	y = 1.234x − 3.393	0.011
60 °C–10 °C	y = 1.465x − 9.062	y = 1.489x − 13.876	y = 1.490x − 17.602	y = 1.463x − 18.126	0.025
70 °C–10 °C	y = 1.811x − 14.357	y = 1.837x − 20.103	y = 1.843x − 24.844	y = 1.794x − 24.417	0.032

**Table 5:** Fitting results of the slope.

	Delay Chain_1	Delay Chain_2	Delay Chain_3	Delay Chain_4	Δ_MAX_
Fitting results	Y = 0.027X − 0.134	Y = 0.028X − 0.136	Y = 0.028X − 0.125	Y = 0.027X − 0.141	0.001
R^2^	0.99848	0.99833	0.99856	0.99862	0.0029

**Table 6:** Resource utilization of a NUMP ^1^ TDC ^2^ implemented in Cyclone 10 LP FPGA ^3^.

	Resources	Used	Utilization
NIOS CPU	logic cells	8826	7.411%
	logic registers	5730	4.812%
	memory bits	104,016	2.612%
NUMP TDC without temperature correction (single channel)	logic cells	1377	1.156%
	logic registers	708	0.595%
	memory bits	23,552	0.592%
Temperature correction module	logic cells	1230	1.033%
	logic registers	689	0.577%
	memory bits	3072	0.077%

^1^ NUMP, non-uniform multiphase; ^2^ TDC, time-to-digital converter; ^3^ Cyclone 10 LP FPGA, Cyclone 10 low power field programmable gate array.
